# Curcumin-Loaded Human Serum Albumin Nanoparticles Prevent Parkinson’s Disease-like Symptoms in *C. elegans*

**DOI:** 10.3390/nano12050758

**Published:** 2022-02-24

**Authors:** Arvie Camille V. de Guzman, Md. Abdur Razzak, Joong Hee Cho, Ji Yi Kim, Shin Sik Choi

**Affiliations:** 1 The Natural Science Research Institute, Department of Food and Nutrition, Myongji University, Yongin 17058, Korea; acvdeguzman.2@gmail.com (A.C.V.d.G.); razzak.abdur@kangwon.ac.kr (M.A.R.); kku1867@gmail.com (J.H.C.); mideum97@gmail.com (J.Y.K.); 2 Department of Energy Science and Technology, Myongji University, Yongin 17058, Korea

**Keywords:** curcumin, human serum albumin, Parkinson’s disease, nanoparticle, dopaminergic neuron

## Abstract

Parkinson’s disease is one of the most common degenerative disorders and is characterized by observable motor dysfunction and the loss of dopaminergic neurons. In this study, we fabricated curcumin nanoparticles using human serum albumin as a nanocarrier. Encapsulating curcumin is beneficial to improving its aqueous solubility and bioavailability. The curcumin-loaded HSA nanoparticles were acquired in the particle size and at the zeta potential of 200 nm and −10 mV, respectively. The curcumin-loaded human serum albumin nanoparticles ameliorated Parkinson’s disease features in the *C. elegans* model, including body movement, basal slowing response, and the degeneration of dopaminergic neurons. These results suggest that curcumin nanoparticles have potential as a medicinal nanomaterial for preventing the progression of Parkinson’s disease.

## 1. Introduction

Parkinson’s disease (PD) is one of the most frequently diagnosed age-related diseases next to Alzheimer’s disease. The prevalence of PD increases as age progresses, and patients are expected to have a decreased life expectancy [1]. PD is known for its motor symptoms, including shaking (both rest and action tremors), stiffness, slower voluntary movements (bradykinesia), and the inability to maintain an upright posture [2]. There is evidence that the onset of motor symptoms is only visible when ~80% of the putaminal dopamine (DA) is diminished and when ~60% of the dopaminergic neurons of the substantia nigra par compacta have been lost [3]. Despite extensive research on PD, there is still no concrete mechanistic explanation as to how neurodegeneration starts. This restricted knowledge is the main hindrance in providing proper neuroprotective therapeutic advancement for PD. However, advancements in bio- and nanotechnologies and the use of natural substances, such as gingko biloba [4,5], ginseng [6,7], and flavonoids [8,9], as novel regimens or supplementary agents are gaining a great deal of interest. In our previous study, we successfully prepared zein-carboxymethyl cellulose (CMC) nanoparticles loaded with dioscin (a steroidal saponin) using an antisolvent precipitation process [10]. DZC nanocomplexes were also used to inhibit dopaminergic neuron degeneration in *C. elegans* successfully, with no specific toxicity being induced.

In the same direction, this study focused on the utilization of curcumin- (CU) loaded nanoparticles as a promising PD therapeutic agent. Numerous studies have suggested its beneficial effects in treating several neurodegenerative diseases [11,12,13,14,15,16,17,18,19,20]. Its health benefits are not only limited to neurodegenerative diseases, and researchers have also discussed numerous biological and pharmacological activities, such as anti-Alzheimer’s, anticancer antibacterial, antioxidant, anti-inflammatory, and anti-tumor activities [21,22]. CU is a polyphenolic compound that is extracted from the rhizomes of a commonly occurring plant known as turmeric (*Curcuma longa*). CU appears as a yellow pigment and is reported to have 3–6% bioactive properties [23,24,25,26,27]. For centuries, it has been popularly used as a food additive and as an herbal medicine in several parts of South Asia [28]. The United States Food and Drug Administration (USFDA) generally regards curcumin as being safe (GRAS) due to its relatively low toxicity [29]. Despite its numerous health benefits, its pharmacological or nutraceutical applications are limited due to its poor oral bioavailability and low water solubility [28]. Various approaches have been applied to enhance the stability and solubility of CU, including encapsulation into proteins, polymeric micelles, and nanoparticles [29,30,31]. Additionally, evidence has shown that the stability of polyphenols such as curcumin can be improved by fabricating a CU–protein complex [32].

HSA, a versatile protein carrier that is extensively used for drug delivery, is an ideal candidate CU–protein nanocomplex fabrication. In the circulatory system, HSA is present at a considerate amount (60%), and it provides 80% of the blood’s osmotic pressure [33]. HSA appears to be globular, composing 585 amino acids in a single polypeptide chain [34]. Research has shown that HSA-based nanocarriers have been proven to enhance the stabilities of certain compounds, especially cancer agents [35,36], while maintaining safe and efficient delivery [37,38,39]. In the present study, we investigated PD alleviation in a *C. elegans* model system using a nanometer-sized particle encapsulating CU with HSA (CUHNP).

## 2. Materials and Methods

### 2.1. Materials

Curcumin (CU) and human serum albumin (HSA) were procured from Sigma-Aldrich Chemical Co. (St. Louis, MO, USA), with no further purification being carried out. Throughout the experiments, analytical grade reagents and double-distilled water were used.

### 2.2. Fabrication of CU-Loaded HSA Nanoparticles (CUHNP)

Lyophilized HSA powder was dissolved in 10 mM pH 7.0 NaCl solution to form a 16 mg/mL HSA aqueous solution. Then, 4 mL absolute ethanol was added to the HSA solution dropwise while being vortexed at a medium speed. The prepared HSA nanoparticle solution was further crosslinked with 48 µL of freshly prepared 8% glutaraldehyde solution. (Commercial 25% glutaraldehyde solution was diluted to 8% using distilled water). To proceed with the crosslinking, the nanoparticle solution was shaken for 15 min. Any insoluble materials left after crosslinking were removed by centrifuging the solution for 10 min at 4500× *g*. The supernatant was removed, and the pellets were washed via dispersal in a 5 mL solution of 10 mM NaCl at pH 7.0 followed by centrifugation at 4500× *g* for 10 min. Washing was carried out at least 5 times to ensure that the glutaraldehyde was removed from the solution. After the washing process was complete, the nanoparticles were filtered using a 0.45 um PVDF syringe filter. The filtered solution was added with an appropriate amount of freshly prepared CU stock solution to achieve the desired concentration of CU in the HSA. 

### 2.3. Fabrication/Characterization of CUHNP

#### 2.3.1. Morphology, Particle Size, PDI, and ζ-Potential

Dynamic light scattering (DLS) was utilized to determine the Z-average diameter and polydispersity index (PDI) of the samples at 25 °C using a He/Ne laser (λ = 633 nm) and a 173° backscatter detector on a Zetasizer Nano ZS (Malvern Instrument, Worcester-shire, UK) [31,32]. 

The ζ-potential of the samples was determined at 25 °C using the Helm–Holtz–Smoluchowski model used for electrophoretic mobility measurements conducted on the same DLS instrument [40,41]. All of the DLS measurements were performed after the ten-fold dilution of the sample in the appropriate pH buffer solution, and data processing was performed using the Malvern Zetasizer Nano-ZS software that was included with the device. All of the measurements were performed at least in triplicate.

The morphology of the freeze-dried samples was examined using a field emission scanning electron microscope (Hitachi SU-70 FE-SEM, Chatsworth, CA, USA). The samples were placed on conductive carbon tape and were platinum-coated using a Hitachi MC1000 Ion Sputter (Hitachi, Singapore) before observation.

#### 2.3.2. Encapsulation Efficiency (EE)

CUHNP solubilization was carried out by stirring the freeze-dried samples (~10 mg) in 10 mL ethyl acetate in the dark overnight. After overnight stirring, the samples were centrifuged for 25 min at 4500× *g*. A ten-fold dilution using ethyl acetate was carried out with the resulting supernatant to reduce the concentration. To assess the CU concentration, the absorbance at 425 nm was obtained by employing a UV/Vis spectrophotometer. The calibration curve was established by employing standard solutions (0–30 µg/mL free CU in ethyl acetate). The encapsulation efficiency (EE) was calculated using:

(1) $$\mathrm{Encapsulation}\;\mathrm{efficiency}\;\left(\%\mathrm{EE}\right)=\frac{\left(\mathrm{total}\;\mathrm{amount}\;\mathrm{of}\;\mathrm{CU}\;\mathrm{added}-\mathrm{free}\;\mathrm{non}-\mathrm{entrapped}\;\mathrm{CU}\right)}{\left(\mathrm{total}\;\mathrm{amount}\;\mathrm{of}\;\mathrm{CU}\;\mathrm{added}\;\left(g\right)\right)}\times 100$$

### 2.4. In Vivo Safety of CUHNP in C. elegans

#### 2.4.1. Nematode Cultivation

*C. elegans* were maintained at standard conditions at 20 °C. Hypochlorite bleaching (1.5% NaOCl and 1.5 M NaOH) was employed to synchronize the worms, and the eggs were subsequently cultured on fresh NGM plates with *E. coli* OP50 [42]. The synchronized worms were grown until the 4th larval (L4) developmental stage, and the larvae were exposed to different diets. L4 stage-synchronized worms were used in all assays.

#### 2.4.2. Analysis of Lifespan 

Lifespan was characterized using an amended methodology determined by Sutphin (2009) [43]. Experiments were carried out on two different set-ups: (1) with control worms that were fed on *E. coli* OP50 and (2) diet-treated worms that were fed with *E. coli* OP50 with added CUHNP (two different concentrations: 10 µg/mL or 35 µg/mL). In each assay, lifespan was scored by tallying the number of surviving and dead animals per day starting from the first day of adulthood. To avoid mixing generations, it was necessary to transfer the nematodes to freshly prepared NGM plates seeded with enough food each day during active reproduction. After the reproductive activities concluded, the nematodes were transferred every three days until all of the worms were dead. The scoring is as follows: (1) nematodes that were stagnant after being prodded gently were tallied as dead, and (2) those that crawled or fled out of the plates were tallied as missing and were subtracted from the lifespan count. Assays were performed in three replicates consisting of three trials per replicate and N ≥ 100 worms per group. Using Kaplan–Meier survival analyses for each group, the cumulative survival patterns were calculated.

#### 2.4.3. Locomotion Assay 

Similar to the lifespan assay, the synchronized L4 worms were raised in two different set-ups: (1) control worms were fed on *E. coli* OP50, and (2) diet-treated worms were fed with *E. coli* OP50 with added CUHNP (two different concentrations: 10 µg/mL or 35 µg/mL). A total of 10 worms were randomly selected from the 24 h, 72 h, and 120 h of adulthood groups (which is equivalent to one, three, and five days of adulthood). Movement scoring was carried out by transferring the randomly selected worms to an unseeded NGM plate. to the plates were tapped gently induce movement stimuli, 2 s were allowed to pass before the bends each worm made were scored. Body bends were counted manually for 60 s. A locomotion assay was performed until the worms reached 5 days of adulthood. Assays were performed in three replicates, consisting of three trials per replicate and had N ≥ 10 worms per group.

#### 2.4.4. Assessment of DA Neuron Degeneration and DA-Related Behaviors

Neurodegeneration assessment was accomplished by raising synchronized L4 BZ555 mutant worms in two different set-ups: (1) control worms were fed on *E. coli* OP50 and (2) diet-treated worms were fed with *E. coli* OP50 with added CUHNP (two different concentrations: 10 µg/mL or 35 µg/mL). Diet exposure took place for 48 h, with a dopaminergic neuron assessment e every 12 h. To assess the neurons effectively, the nematodes were sandwiched in 2% agarose pads with a drop of 5 M levamisole being previously added and coverslip on top). Before sandwiching, the worms were washed with an M9 buffer to eliminate the bacterial debris clinging to the body. An amount of 5 M Levamisole was added to aid in the immobilization of the worms [44]. Neurodegeneration monitoring was carried out by imaging the living (immobilized) worms’ dopamine neurons tagged with green fluorescence (GFP). The assay was carried out using a fluorescence microscope, Axio Imager A2 (Carl Zeiss, Jena, Germany) at a fixed fluorescence exposure time. Each photographed image was analyzed using ImageJ software. 

#### 2.4.5. Assessment of DA-Related Behaviors

##### Basal Slowing Response (BSR) Assay

BSR was explored by employing a slightly modified protocol described by Chase [45]. The assay was accomplished by separately raising well-fed synchronized L4 N2 and *cat-2*-defective mutant worms. Each strain was raised in two different set-ups: (1) control worms were fed OP50 and (2) diet-treated worms were fed with OP50 with added CUHNP (two different concentrations: 10 µg/mL or 35 µg/mL). A total of 30 individual worms from each set-up were randomly chosen and were transferred to separate freshly prepared unseeded NGM plates for exactly 5 min. After 5 min, the worms were divided equally and were moved to two separate plates labeled: (1) “with-food plate” (NGM media with seeded OP50) and (2) “no-food plate” (unseeded NGM media). Before scoring the body movements, the worms were incubated for 2 min, allowing them to acclimatize first. The body bends were counted manually for 20 s. Percent basal slowing was calculated using the formula:

(2) $$\mathrm{BSR}=\frac{{\mathrm{rate}}_{\mathrm{on}\;\mathrm{food}}-{\mathrm{rate}}_{\mathrm{off}\;\mathrm{food}}}{{\mathrm{rate}}_{\mathrm{off}\;\mathrm{food}}}$$

#### 2.4.6. Ethanol Avoidance Test 

Similar to the BSR assay, the ethanol avoidance test was accomplished by separately raising synchronized well-fed L4 stage N2 and cat-2 defective mutant worms. Each strain was raised in two different set-ups: (1) control worms were fed on OP50 and (2) diet-treated worms were fed OP50 with added CUHNP (two different concentrations: 10 µg/mL or 35 µg/mL). The test plate assay was carried out using unseeded NGM plates that were divided into four equal quadrants. At the center of the plate, a 1 cm-diameter circle was marked. Total amount of 4 µL of ethanol and water were spotted 2.5 cm away from the center and were positioned diagonally from each other. In the initially marked center, a randomly selected 10 synchronized L4 worms were spotted and were allowed to roam around. Before the worms were place in the center, the nematodes were thoroughly washed using M9 buffer to remove any E. coli OP50 debris. Ethanol avoidance was carried out by tallying the number of animals in both the ethanol and water quadrants, and the preference index (PI) was calculated using the following formula:

(3) $$\mathrm{Preference}\;\mathrm{index}=\frac{{\mathrm{number}\;\mathrm{of}\;\mathrm{worms}}_{\mathrm{ethanol}\;\mathrm{quadrants}}-{\mathrm{number}\;\mathrm{of}\;\mathrm{worms}}_{\mathrm{control}\;\mathrm{quadrants}}}{\mathrm{total}\;\mathrm{number}\;\mathrm{of}\;\mathrm{worms}\;\mathrm{tested}}$$

## 3. Results

### 3.1. Fabrication/Characterization of CU-Loaded HSA Nanoparticles (CUHNP)

#### 3.1.1. Effect of CU Concentrations on Particle Size and ζ-Potential

**Figure 1:**
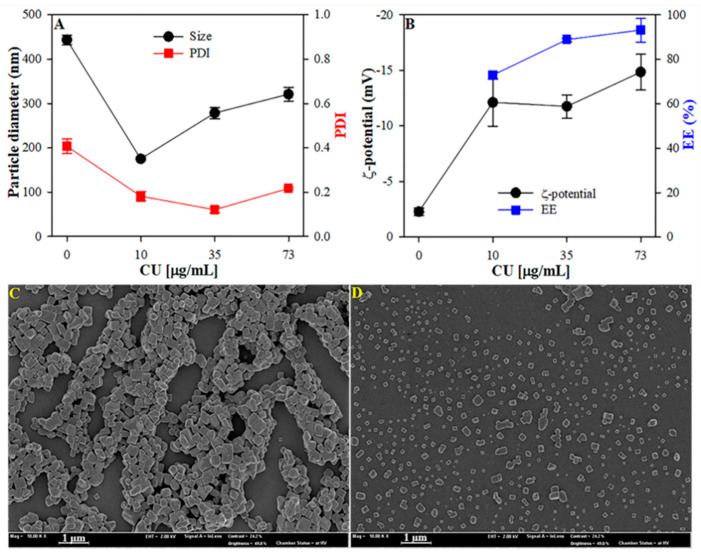
Fabrication of stable and homogeneous CUHNP. Particle size, PDI (**A**), ζ-potential, and encapsulation efficiency (**B**) of CUHNP as a function of CU concentration and SEM images of HSA (**C**) and CUHNP (**D**) nanoparticles.

The preparation of the HSA nanoparticles can be achieved through the desolvation method, in which the desolving agents such as alcohol are added dropwise followed by the addition of a crosslinking agent, glutaraldehyde. Then, curcumin can be successfully incorporated by adding it directly to the formed HSA nanoparticles while mixing [46]. Figure 1 depicts the influence of increasing the CU concentration on the particle size, PDI, and ζ-potential of complex nanoparticles. The particle size of bare HSA nanoparticles was ~435 nm, with a PDI value of 0.04 (Figure 1A). After adding 10 µg/mL of CU, the particle size and the PDI value of the nanoparticles decreased by two-fold, from ~435 nm to ~200 nm and from 0.04 to 0.22, respectively. However, a concentration-dependent increase in the particle size was noted while maintaining the PDI after adding more CU to the nanoparticle. Changes in the ζ-potential values could help to further explain the particle size results (Figure 1B). The ζ-potential of plain HSA was ~−2.5 mV. The ζ-potential of complex nanoparticles increased from −2.5 to −13 mV.

Nanoparticle-mediated delivery systems have been demonstrated to improve the oral bioavailability of this nonpolar bioactive compound [47,48]. To evaluate the encapsulation of curcumin (CU) molecules into complex nanoparticles, UV/Vis spectroscopy was utilized. As shown in Figure 1B, the encapsulation efficiency was as high as 77% when a small concentration of curcumin was added to the nanoparticles. Remarkably, the EE of CU reached 93% after adding a higher concentration of CU. These results along with the particle sizes revealed that CU was effectively integrated into the HSA nanoparticle.

#### 3.1.2. Morphology of CUHNP 

FE-SEM was used to observe the structure and surface appearance of the HSA nanoparticles in the absence and presence of CU, as shown in Figure 1C,D. HSA was observed to have a cube-shaped flaky microstructure. The presence of curcumin resulted in an individual particulate structure. The particulates were mostly cube-shaped and had smooth surfaces.

### 3.2. In Vivo Assessment of CUHNP in C. elegans

#### 3.2.1. Enhanced Lifespan after Feeding with CUHNP

CU and HSA are already considered GRAS and have been widely used in several therapeutic applications, although their nanoparticle transformation requires additional safety confirmations. In these experiments, the in vivo safety of CUHNP was confirmed using *C. elegans* models. The in vivo testing involved lifespan and body movement measurements. 

**Figure 2:**
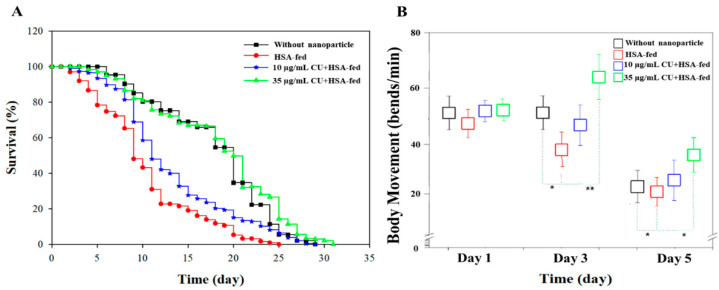
Biological safety of CUHNP in *C. elegans*. Lifespan (**A**) and body movement (**B**) of *C. elegans* that were cultured without nanoparticles, with HSA, and with 10 µg/mL and 35 µg/mL of CU-loaded HSA nanoparticles (CUHNP or CU + HSA), respectively. The data present the mean ± S.D. from three independent experiments (*p* < 0.01).

To determine the effect of the CUHNP, two different concentrations (10 µg/mL and 35 µg/mL) were introduced to *C. elegans*. For re-confirmation, an empty HSA-NP was also fed to the animals. The effect of each additional diet was calculated using Kaplan–Meier survival analysis with a Log-rank significance test. From Figure 2A, the survival ratio of the animals fed HSA exhibited a slight decrease, resulting in a shortened mean lifespan of 24.33 ± 2.08 days, although this decrease is not statistically different from the survival ratio of the control worms (without nanocomplex) of 28.00 ± 0.79 (*p* < 0.001). This observation only suggests that the cross-linking step using glutaraldehyde resulted in an acceleration of the aging process; hence, additional washing steps were carried out to ensure the complete removal of the excess glutaraldehyde. Interestingly, this reduction in the lifespan was successfully recovered when the bare HSA nanoparticles were added along with the core compound, curcumin. The calculated mean lifespans for the worms that had been treated with 10 µg/mL and 35-µg/mL were 28.33 ± 1.53 and 31.0 ± 1.57, respectively. The overall results indicate that the oral administration of CUHNP is considered safe in terms of the survival ratio over the course of the entire lifespan of *C. elegans*, showing that no significant differences are observed in the CUHNP group compared to the control group worms (Figure 2A).

#### 3.2.2. Enhanced Body Movement after Feeding with CUHNP

A lifespan assay is not enough to establish the biological safety of the CUHNP, so its effect on body movement was also determined. The validation of the effect of CUHNP was carried out by measuring the body bends on the first, third, and fifth days of adulthood. Interestingly, on the third day of adulthood, the nematodes that had been fed with the different diets, HSA nanoparticles only or 10 µg/mL and 35- µg/mL of curcumin, exhibited a significant decline or enhancements in their body movements, even if their survival ratio was similar to the control ones. As expected, worms that had been treated with HSA nanoparticles only exhibited a decrease in body movement that coincided with the lifespan assay results. However, 35 µg/mL of CUHNP effectively delayed the age-related deterioration of movement on the third day of adulthood (Figure 2B) from 35 bends/min to 65 bends/min. 

As discussed above, the aging or the development of neurodegenerative diseases is linked with changes in movement behaviors [49]. Taking into account that the CUHNP aided in the enhancement of body movements, these nanoparticles might have the potential to delay neurodegeneration. The next approach aimed to confirm the effectiveness of the CUHNP on the dopamine-related behavior of *C. elegans.*

### 3.3. Improvement of DA-Regulatory Behaviors by CUHNP

#### 3.3.1. Basal Slowing Response (BSR) Assay

To further assess the effect of the CUHNP on DA-related behavioral movements in *C. elegans*, a comparison of the basal slowing response (BSR) and ethanol avoidance between N2 worms and dopamine-deficient mutant strains, *cat-2*, both of which had been fed with and without the nanoparticles, was performed. 

**Figure 3:**
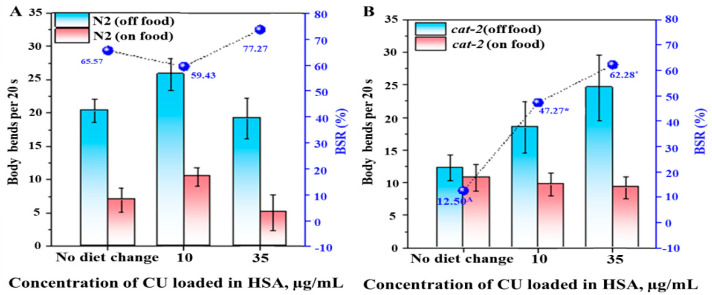
Improvement in DA-regulated behavior, BSR, in *C. elegans* by CUHNP. Body movement (left y-axis, bends per 20 s) and basal slowing response (BSR) percentage (right y-axis) of wild-type N2 (**A**) and *cat-2*(e1112) mutant (**B**) *C. elegans* fed without nanoparticles and with 10 µg/mL and 35-µg/mL CU-loaded HSA nanoparticles (CUHNP), respectively. The data present the mean ± S.D. from three independent experiments (*p* < 0.01). * means exhibited enhanced BSR within worm strain group, and ^A^ represents a defect in BSR compared to in wild type samples.

The BSR assay is a behavioral test that assesses worm behavior when they are placed on an agar plate in the presence and in the absence of bacterial food. BSR is mainly executed by the DA neuron system; hence, any defect in the DA neuronal system will result in a defective BSR, as observed from the cat-2 mutant worms [45]. As expected, the wild-type nematodes exhibited slower locomotion when placed on the (+) food plates (≈7 bends/20 s) compared to the worms on the (−) food plates (≈20 bends/20 s) (Figure 3A). Since the wild-type N2 worms exerted intact DA signaling, no significant differences in the BSR behavior were displayed between animals who received 10 µg/mLand 35-µg/mL of CUHNP and the control worms (no nanoparticles) (≈59–80%) (Figure 3A). These results indicate that there is no toxic effect induced that is by the CUHNP on DA neuron-governed behavior, BSR.

On the other hand, *cat-2* mutants have a defective DA biosynthetic process due to the lack of ortholog in the human tyrosine hydroxylase (TH) gene. This disables the ability of *cat-2* mutants to recognize new surroundings and does not reduce their movement speed as if they are still in space without food. The control group of the *cat-2* mutants (without exposure to CUHNP) displayed a very low BSR of ~12.5%. The low BSR percentage was rescued after the *cat-2* worms were exposed to 10 µg/mL and 35-µg/mL CUHNP, displaying a significant reduction (*p* < 0.01) in their movement speed in the new plate containing food, resulting in ≈50–60% BSR. The change in the BSR percentage was more pronounced in the animals fed with 35-µg/mL CUHNP, and this is almost similar to the case of the wild-type N2 strain (Figure 3B). These results demonstrate that CU delivery can be successfully attained by employing an HSA nanoparticle vehicle. The results from the *cat-2* experiments claim that the CUHNP can be orally administered to turn on alternative DA-like neurotransmitter synthesis or to enhance the efficiency of DA transmission. 

#### 3.3.2. Ethanol Avoidance

For an additional confirmatory DA-regulated behavior assay, an ethanol avoidance test was also performed. In this assay, the worms were not exposed to nor fed ethanol; instead, healthy and well-fed worms were exposed to ethanol for 10 min in order to observe their chemotactic behavior (attraction/repulsion). As expected, worms with the same intact DA signaling as that in the wild-type N2 exhibited a 100% alcohol avoidance ratio during day 1 of adulthood (Supplementary Figure S3). The alcohol avoidance ratio in the N2 worms who did not receive nanocomplex exposure was diminished at day 2 of adulthood (≈60%); however, such a reduction in alcohol avoidance at day 2 was recovered in the worms that had been fed 35 µg/mL of CUHNP (≈100%). The wild-type N2 worms that had been fed10 µg/mL of CUHNP exhibited an 80% avoidance at day 2. In the cat-2 context, the effect of both nanoparticle concentrations was dramatically exerted, resulting in ≈100% alcohol avoidance; however, none of the worms cultured in the absence of the nanocomplex showed alcohol avoidance (≈−30%). These results demonstrate that there might be *cat-2*-independent pathways for the execution of DA-related behaviors.

### 3.4. Prevention of Dopaminergic Neuron Degeneration by CUHNP

Similar to our previous study on the DZC nanocomplex [10], one feasible explanation for the beneficial effect of CUHNP on DA-regulated behaviors is that nanoparticles might promote DA transportation. In *C. elegans*, the DAT-1 gene encodes the DA transporter which is expressed in two pairs of anterior (ADE, CEP) and one pair of posterior (PDE) DA neurons [50].

**Figure 4:**
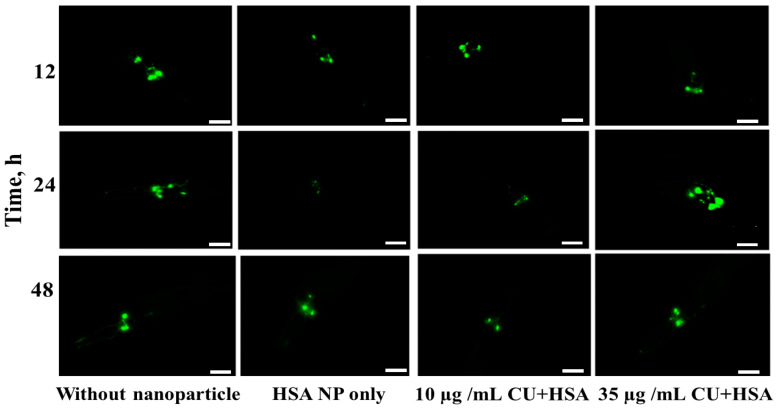
Protection of DA neurons against neurodegeneration. Fluorescence images of anterior DA neurons of BZ555 [dat-1::gfp] *C. elegans* strain fed with and without CU-loaded HSA nanoparticles (CUHNP) at 12 h, 24 h, and 48 h of adulthood, respectively. Scale bar: 50 µm.

**Figure 5:**
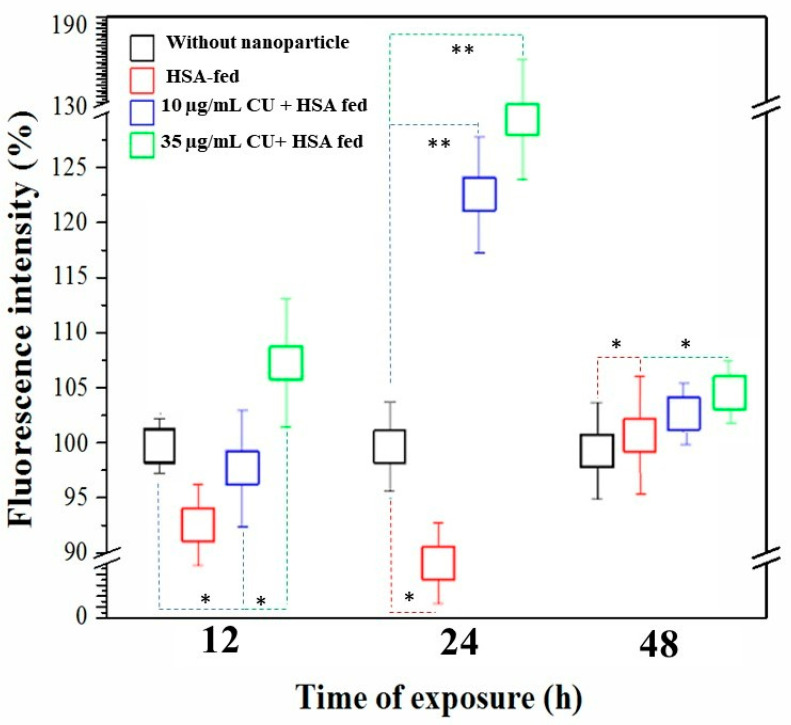
Computation image analysis of the fluorescence intensities after CUHNP treatment. Fluorescence intensity of DAT-1::GFP expressed in DA neurons significantly protected by 35 µg/mL of CU-loaded HSA nanoparticles (CUHNP) after 24 h of exposure. * (*p* < 0.01) ** (*p* < 0.001).

To confirm whether the effect of the CUHNP can actively promote DA transport, the use of the Pdat-1::GFP transgenic strain, BZ555, was employed. Compared to the day 1 adult worms maintained with different diets, the DA transporter, DAT-1, was more expressed or activated in the animals fed 35 µg/mL CUHNP, showing brighter green fluorescence ranging from the GFP tagged to DAT-1 groups (Figure 4). Based on the computation image analyses (Figure 5), the fluorescence intensity of the CUHNP-fed group within 12 h is approximately 107% stronger than that of the control groups (without CUHNP). The said increase in the DAT-1 activity was more pronounced after the worms had been the CUHNP diet for 24 h, resulting fluorescence intensity that was about 130% stronger. These results provide evidence that the CUHNP can improve the activity of DA transporters at the end of presynaptic neurons, resulting in enhanced DA transportation to synaptic neurons. 

## 4. Discussion

Aging is an inevitable phenomenon in every living organism. Additionally, the prevalence of Parkinson’s disease (PD) increases as humans become older. Despite extensive research about PD, there are still no medications for it. Because of this, the study employed a *C. elegans* model system to assess the effect of functional food nanocomplexes for PD prevention. 

Several naturally occurring compounds are known to be beneficial in preventing PD progression, including gingko biloba [4,5], ginseng [6,7], flavonoids [8,9], dioscin [10], and curcumin [47]. Curcumin is known for various biological activities; however, its pharmacological or nutraceutical applications are limited due to its poor oral bioavailability and low water solubility [28]. Curcumin is readily soluble in ethanol; however, the use of such solvents can induce negative effects when ingested directly by the nematodes [51]. In this study, the effects of fabricating a human serum albumin (HSA)-based nanocarrier for curcumin to enhance its neuroprotective effects were assessed. HSA was specifically chosen because of its high biodegradability and because it does not induce any serious side-effects, as proven by numerous clinical studies [52,53]. HSA-based nanoparticles can be prepared by employing the desolvation method, where a desolving agent is added dropwise to the aqueous albumin solution, followed by the addition of a crosslinking agent [54,55]. Crosslinking is an important step that can affect the release of the compound from the system [56], and the most commonly used stabilizer or crosslinking agent is the glutaraldehyde (GA) [57,58]. Following this method, curcumin (CU) was successfully encapsulated with an HSA nanoparticle, having a very high EE of 90%. Based on our previous reports, curcumin’s aqueous solubility was successfully enhanced by 234-fold when using the albumin–protein nanocarrier (ovalbumin), while curcumin degradation in aqueous solutions was significantly diminished (0.25-fold) at a wide range of pHs (1.2–8.5) [59]. Thus, it can be hypothesized that a small molecule such as curcumin would be readily released from HSA nanoparticles in vivo and would have the intended effects, which were substantiated by our findings.

From the lifespan assay we performed, a slight decrease in the lifespan of the worms was noted after feeding with HSA-NP, but this decrease is not significantly different from the control group. The bare HSA-NP is only composed of the albumin and the crosslinker, GA. Although some research has reported that GA exhibits some toxicity [60,61], we can rule out the toxicity effect since the worms are still alive throughout their expected average lifespan. In fact, the observed mean lifespan of the HSA-fed worms is still 2 days longer compared to our previous nanocomplex (dioscin-loaded zein–CMC nanocomplexes), where a mean lifespan of 22.33 ± 2.08 days was observed [10]. Moreover, the results also showed that the addition of curcumin to the bare HSA-NP rescued the lifespan. This further confirms that curcumin was successfully introduced to *C. elegans*. The oral supplementation of curcumin-loaded HSA nanoparticles was found to prolong the lifespan of the worms more efficiently, by 2–3 days. Worms are expected to complete their full lifespan in a span of a month; hence, the 4–5-day difference in the lifespan portrayed by the HSA-fed animals only shows an advanced aging process for the worms. This result was further supported by the observed enhanced movement, DA-related behaviors as well as the protection of the DA neurons.

Examining the DA synthesis pathway in *C. elegans,* dopamine is mostly synthesized from the precursor tyrosine (Tyr) with the aid of the catalyzing agent tyrosine 3-monooxygenase-forming levodopa (L-DOPA), which is further decarboxylated by the amino acid decarboxylase (AADC) [49,62]. In the case of *cat-2* mutant worms, they do not carry the genes responsible for encoding the tyrosine hydroxylase, making this mutant incapable of synthesizing DA compared to the N2. However, the major results of the study showed that the *cat-2* mutant worms significantly exhibited an improvement in DA-related behaviors, including in the BSR percentage and alcohol avoidance. The response time of the worm to volatile repellent ethanol is an indicator of the DA levels. With a normal amount of DA, the worm immediately repels away from the ethanol scent by moving away from it, while worms with a decreased DA content does not recognize the ethanol scent and tends to stay in the zone [63]. The repulsion behavior that we observed indicated that curcumin offered almost the same DA content as that seen in the control worms. The same was observed in the BSR assay, which implies that curcumin contributed to dopamine supplication. This is further supported by the conservation of the DA neurons observed on the BZ555 mutant worms. 

In conclusion, combining all the results, we propose that the activity of DAT-1, a DA transporter, can be enhanced through CU supplementation in the diet. CUHNP supplements can act as a DA transport, contributing to neuronal signaling and controlling behaviors. CUHNP was found to be beneficial in protecting DA neurons inhibiting the manifestation of Parkinson’s disease-like symptoms in a *C. elegans* PD model system. 

## Data Availability

The data described in this research are accessible from the corresponding author upon request.

## Supplementary Materials

The following supporting information can be downloaded at: https://www.mdpi.com/2079-4991/12/5/758/s1, Figure S1: FTIR spectra; Figure S2: Pharyngeal pumping of the *C. elegans* [64]; Figure S3: Enhanced ethanol avoidance.
