# Supplementary material for: Curcumin-Loaded Human Serum Albumin Nanoparticles Prevent Parkinson’s Disease-like Symptoms in C. elegans
Source: Nanomaterials (Basel). 2022 Feb 24;12(5):758. doi: 10.3390/nano12050758 (PMC8924894; doi:10.3390/nano12050758)

Supplementary Information

**Curcumin-loaded Human Serum Albumin Nanoparticles Pre-vent Parkinson's Disease-like Symptoms in C. elegans**

Arvie Camille V. de Guzman [1], Md. Abdur Razzak [1, 2], JiYi Kim[1], and Shin Sik Choi [1, 3*]

[1]Natural Science Research Institute, and Department of Food and Nutrition, Myongji University, Yongin, Republic of Korea; acvdeguzman.2@gmail.com (AC.V.DG); razzak.abdur@kangwon.ac.kr (MA. R); mide-um97@gmail.com (JY. K)

[2]Kangwon Institute of Inclusive Technology (KIIT), and Department of Food Science and Biotechnology, College of Agriculture and Life Sciences, Kangwon National University, Chuncheon-si, Gangwon-do, Republic of Korea.

[3]Department of Energy Science and Technology, Myongji University, Yongin, Republic of Korea.

* Correspondence: sschoi@mju.ac.kr1

# 1. Experimental Section

## 1.1. CU and HSA interaction analyzed through FTIR

The possible interactions between CU and HSA were assessed by employing FTIR (Fourier transform infrared) spectroscopy via the ATR method by employing an Agilent Cary 360 spectrometer at 25 ºC. Freeze-dried CUHNP samples were placed between the diamond crystal and the ATR accessory. A physical mixture of CU and HSA was also subjected to FTIR to see how nanoparticle formation differs from that of physical mixing. The spectra ranged from 400 to 4000 $cm^{-1}$.

## 1.2. Assessment of the worms' intake of CU loaded HSA nanoparticles

A simple way to measure successful feeding is to count the feeding rate of C. elegans. The feeding rate is directly proportional to pharyngeal pumping, which can be directly quantified by counting pumpsper minute using a microscope, Axio Imager A2 (Carl Zeiss, Jena, Germany).

## 2. Results

**Supplementary File Figure S1—Fourier transform infrared spectroscopy (FTIR) spectra of CU, HSA, and CU-HSA physical mixtures and CU-HSA nanoparticles (CUHNP).**

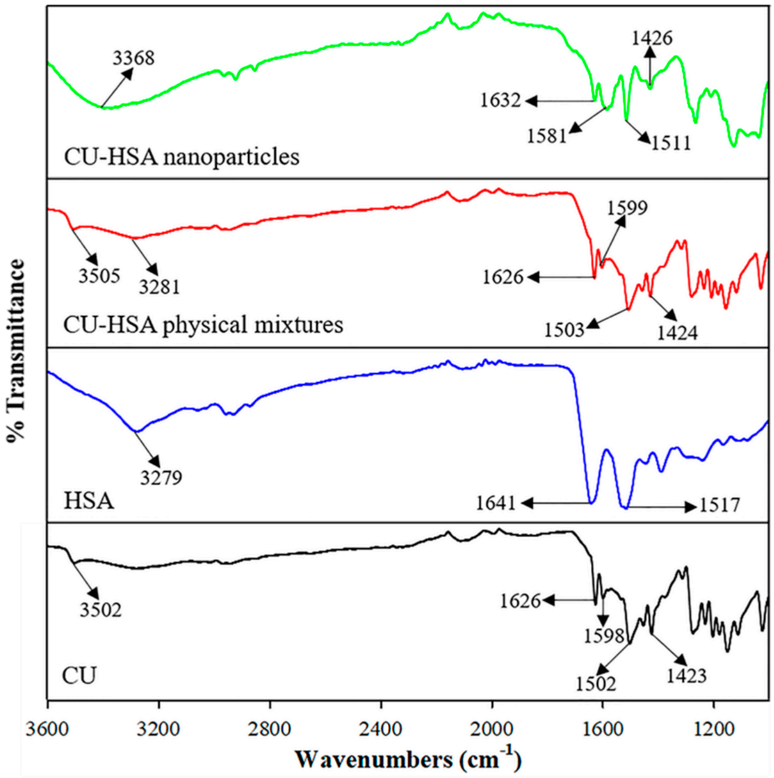


FTIR was employed to identify the forces responsible for the formation of CUHNP nanoparticles. The spectra of CU alone showed characteristic bands at 3502, corresponding to the phenolic O-H stretching vibration. In addition, aromatic C=C band stretching was also visible at 1626 $cm^{-1}$ and 1595 $cm^{-1}$.

For the HSA, a peak appeared at 3279 $cm^{-1}$ that also corresponds to O-H. The formulated CUHNP demonstrated an O-H bond at 3420 $cm^{-1}$ that is slightly stronger than curcumin alone.

**Supplementary File Figure S2—Pharyngeal pumping of the *C. elegans*.** Wild type N2 worms exhibited no significant changes in their pharyngeal pumping, even after being fed the nanocomplex. (*** p<0.001) N=15 worms.

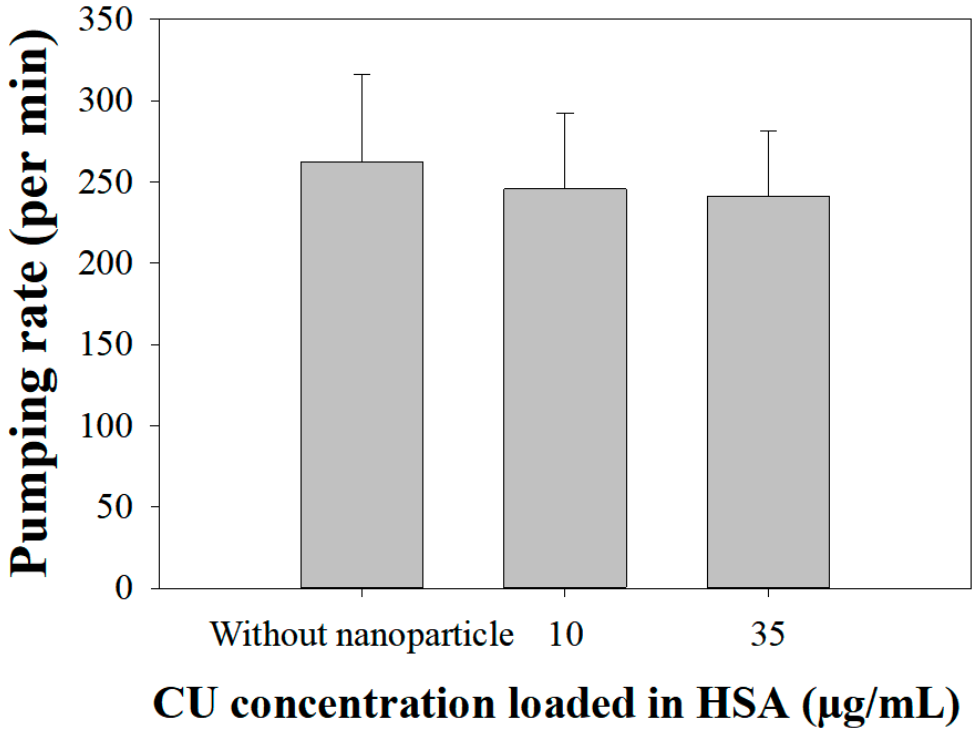


*C. elegans* are able to intake food particles that are about 0.5um in diameter due to their pharyngeal structures [64]; hence, larger curcumin aggregates might not be able to enter their pharynx. Upon feeding the worms with ~200 nm size of CUHNP, there were no changes in the pharyngeal pumping rate from the control group, which means that *C. elegans* were able to intake the CU-loaded HSA nanoparticles that had been added to their diet.

**Supplementary File Figure S3—CUHNP consumption resulted in enhanced ethanol avoidance.** Wild-type N2 worms exhibited no significant changes in ethanol avoidance, even after being fed the nanocomplex (A). The *cat-2 (e1112)* mutant worms, on the other hand, exhibited rescued alcohol avoidance after treatment with CUHNP. (*** $p<0.001$)

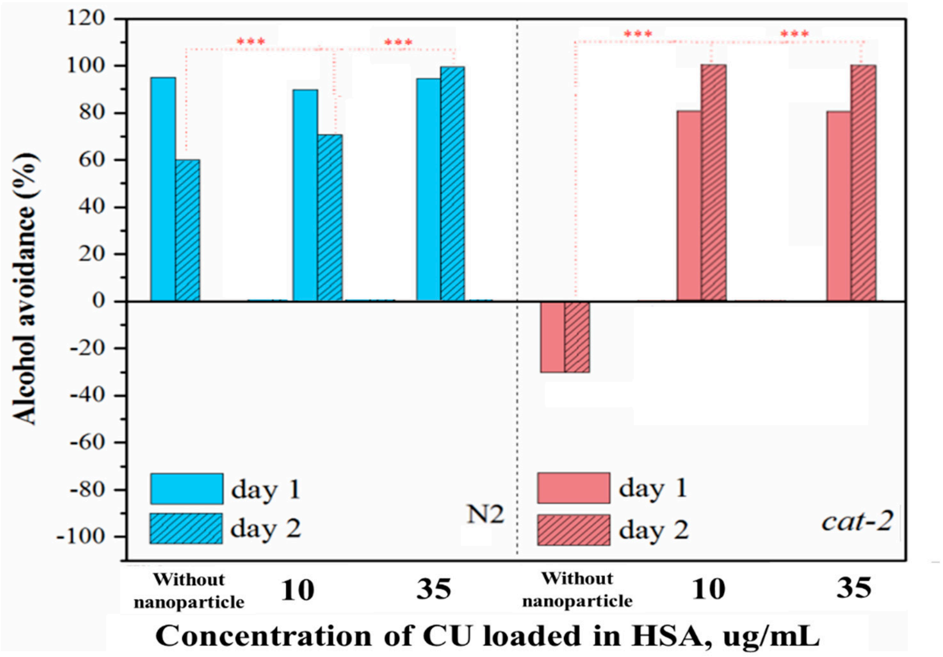

Supplement: Supplementary file 1 [file nanomaterials-12-00758-s001.zip › nanomaterials-1560640-supplementary.pdf]
